# Research trends and hotspots of circular RNA in cardiovascular disease: A bibliometric analysis

**DOI:** 10.1016/j.ncrna.2024.04.002

**Published:** 2024-04-16

**Authors:** Zehui Xu, Chong Guan, Ziji Cheng, Houle Zhou, Wanting Qin, Jiaming Feng, Melisandre Wan, Yihan Zhang, Chengyao Jia, Shuijin Shao, Haidong Guo, Shaoling Li, Baonian Liu

**Affiliations:** a Department of Anatomy, College of Chinese Integrative Medicine, Shanghai University of Traditional Chinese Medicine, Shanghai, 201203, China; b School of Rehabilitation Science, Shanghai University of Traditional Chinese Medicine, Shanghai, 201203, China; c TCM Regulating Metabolic Diseases Key Laboratory of Sichuan Province, Hospital of Chengdu University of Traditional Chinese Medicine, Chengdu, 610072, China; d Guanghua School of Stomatology, Sun Yat-Sen University, Guangzhou, 510080, China; e Department of Pathology, Shanghai Pulmonary Hospital, School of Medicine, Tongji University, Shanghai, 200433, China

**Keywords:** Circular RNAs, Cardiovascular diseases, Biomarker, Therapeutic target

## Abstract

From a global perspective, cardiovascular diseases (CVDs), the leading factor accounting for population mortality, and circRNAs, RNA molecules with stable closed-loop structures, have been proven to be closely related. The latent clinical value and the potential role of circRNAs in CVDs have been attracting increasing, active research interest, but bibliometric studies in this field are still lacking. Thus, in this study, we conducted a bibliometric analysis by using software such as VOSviewer, CiteSpace, Microsoft Excel, and the R package to determine the current research progress and hotspots and ultimately provide an overview of the development trends and future frontiers in this field. In our study, based on our search strategy, a total of 1206 publications published before July 31, 2023 were accessed from the WOSCC database. According to our findings, there is a notable increasing trend in global publications in the field of circRNA in CVDs. China was found to be the dominant country in terms of publication number, but a lack of high-quality articles was a significant fault. A cluster analysis on the co-cited references indicated that dilated cardiomyopathy, AMI, and cardiac hypertrophy are the greatest objects of concern. In contrast, a keywords analysis indicated that high importance has been ascribed to MI, abdominal aortic aneurysm, cell proliferation, and coronary artery diseases.

## Highlights

• CircRNAs in CVDs have been an active research interest.
• CircRNAs in dilated cardiomyopathy, AMI, and cardiac hypertrophy are with greatest concern.
• The role of circRNAs in CVDs and its molecular mechanism are the current research orientation.
• The potential of circRNAs as therapeutic targets or remedies for CVDs is the research hot spot.
• CircRNAs translating polypeptides hasn't yet been widely studied in CVD, holding great value to be further discussed.

## 1 Introduction

Sanger et al. were the first to discover covalently closed circular RNA (circRNA) in 1976. Correlated studies were subsequently conducted, but their scope was constrained by the limitations of sequencing technology [1]. It was not until 2010 that circRNAs became a research focus again and a significant number of related studies were conducted. CircRNAs are covalently closed, single-stranded RNAs (ssRNAs) deriving from pre-mRNA backsplicing [2], during which 3′ and 5’ phosphodiester bonds are formed [3]. The closed-ring structure provides circRNAs with the capacity to resist degradation by RNase R enzymes [4]; thus, it exhibits greater stability than homologous mRNA [5,6]. In addition, these molecules are extensively detected in human tissues [7,8], especially in the brain [9,10], and their expression displays evident specificity in tissues, embryonic stages, and diseases [[11], [12], [13]]. Moreover, by binding with miRNAs [14,15], interacting with proteins [[16], [17], [18]], and competing with endogenous mRNAs [19,20], circRNAs play an indispensable part in the modulation of cellular processes, both physiologically and pathologically. The latest research has also proven that circRNAs can play a role in coding and translating peptides [[21], [22], [23], [24], [25]]. At present, the role of circRNAs has been evidenced in various diseases, for instance, cancer [[26], [27], [28]] and cardiovascular diseases [29], which have received increasing attention. Most importantly, studies have reported the potential of circRNAs in serving as diagnostic biomarkers [30,31] as well as a therapeutic strategy [32,33] in diseases.

As the World Health Organization proclaimed, in 2021, that CVDs represent a major population mortality factor that should be urgently addressed, the role and mechanisms of circRNAs in CVDs have been increasingly studied and have become the latest research hotspot [34]. Firstly, newly conducted studies on circRNAs have provided references for the possible risk of CVD onset. Liu et al. identified 485 circRNAs with varying expression in the aortic vascular tissues of spontaneously hypertensive rats (SHRs); by comparing them with findings in Wistar Kyoto (WKT) rats, they hypothesized and demonstrated that three circRNA–miRNA–mRNA axes in the aorta of SHRs regulated the function of the NOTCH1, FOXO3, and STAT3 genes, respectively [35]. Another study showed that circRNA_0037911 and circRNA_0126991 were upregulated and circRNA_0005870 downregulated in the peripheral blood of patients with hypertension, thus demonstrating that these circRNAs have great potential for becoming biomarkers of this condition [36]. Furthermore, the study progress in circRNAs has provided the basis for the research and development of therapeutic drugs for cardiovascular diseases. For instance, the cardiac regenerative effects of circHIPK3 were identified by Si et al. in their study [37]. Once overexpressed, circHIPK3 can enhance the proliferation, migration, and angiogenesis of coronary vascular endothelial cells and can further improve cardiac dysfunction and decrease the fibrotic area associated with myocardial infarction; therefore, this molecule is believed to be a novel therapeutic target in myocardial infarction. An increasing number of studies have been carried out and confirmed that circRNAs have an enormous impact on the molecular mechanism of various diseases, from pathogenesis to progression.

Bibliometrics is a discipline that takes literature systems and bibliometric characteristics as the subject of study, and quantitative and qualitative analyses of the literature are conducted in order to summarize previous research results and provide suggestions for future investigations [38]. Bibliometric analysis is of great avail to the assessment of research over time, both statistically and qualitatively. It has also been applied in data examination, from online bibliographic databases to metrological characterizations [39]. In bibliometrics, the output and contribution of individuals, institutions, or national support in a given field are measured and evaluated by using relevant parameters, such as the number of published articles, impact factors, and citation rate [40]. Bibliometric studies are informative and useful for the development of research in various fields.

Since 2017, research focused on the application of circRNAs in cardiovascular diseases has increased significantly, but quantitative studies in these overlapping fields are still scarce. In this study, we conducted a mathematical and statistical analysis of articles related to the application of circRNAs in CVDs. By assessing the results and impact of related studies conducted in the last decade from a bibliometric perspective, we summarized the latest progress in the field, identified research hotspots, and discussed future development directions.

## 2 Methods

### 2.1 Searching strategy and data collection

**Fig. 1:**
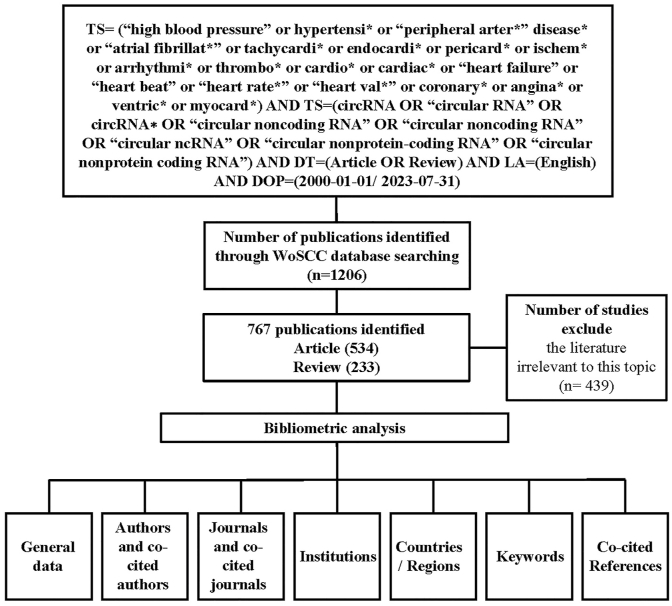
Flowchart of the literature selection.

The Web of Science database (https://login.webofknowledge.com/) was used to implement our search for publications and data collection, which were accomplished on August 2, 2023. All publications were output in the format of “Full Record and Cited References” as plain text files. The following search formula was applied: TS= (“high blood pressure” or hypertensi* or “peripheral arter*” disease* or “atrial fibrillat*” or tachycardi* or endocardi* or pericard* or ischem* or arrhythmi* or thrombo* or cardio* or cardiac* or “heart failure” or “heart beat” or “heart rate*” or “heart val*” or coronary* or angina* or ventric* or myocard*) AND TS=(circRNA OR “circular RNA” OR circRNA∗ OR “circular noncoding RNA” OR “circular noncoding RNA” OR “circular ncRNA” OR “circular nonprotein-coding RNA” OR “circular nonprotein coding RNA”) AND DT=(Article OR Review) AND LA=(English) AND DOP=(2000-01-01/2023-07-31). Two investigators (Zehui Xu and Houle Zhou) retrieved and filtered the publications. Disagreements were discussed with the corresponding authors until a consensus was reached (Fig. 1).

### 2.2 Data standardization

Keywords were standardized to avoid meaningless repetition in the keyword co-occurrence graph, which may result from inconsistency in pos, as well as plural and singular versions of the same keyword. Countries/regions were also standardized; for instance, England, Scotland, Wales, and Northern Ireland were merged into the United Kingdom, and Taiwan was merged with China.

### 2.3 Visualization analysis

The software applied in our study was as follows: VOSviewer (version 1.6.18; Leiden University, Leiden, Netherlands), CiteSpace (version 6.2. R4; Chaomei Chen, Drexel University, Philadelphia, PA, United States), Microsoft Excel (Redmond 2019; WA, United States), R language-based Bibliometrix Package (4.1.3 Package), and Scimago Graphica (Beta 1.0.36). These were used to analyze the data, and the results were exported to a table summarizing the bibliometric parameters, including publication count and year, total and average citation number, title, country and institution, authors, journal, keywords, and references. The maps of co-occurrence of countries/regions were generated with VOSviewer and Scimago Graphica and those of co-occurrence and cooperation among institutions with VOSviewer. In VOSviewer maps, the nodes that corresponded with weights indicated collaborative relationships or co-appearances in a single piece of literature, whereas link thickness was positively correlated with link strength. In the module visualization network, colors were used to differentiate clusters, while in the overlay visualization module, node colors were used to represent the average publication year, where blue represents the previous research phase and yellow the current period. We used CiteSpace to produce a keyword burst map and to identify the references, where dark and light nodes indicate earlier and recent publications, respectively. Furthermore, keywords and references were sorted according to the first year of the burst period in the burst module. R language-based Bibliometrix Package (4.1.3 Package) was used to analyze three-field plots, thereby demonstrating the relationships among co-cited references, authors, and keywords.

## 3 Results

### 3.1 Analysis of the publication and citation trends

A total of 1206 publications were originally retrieved based on our search strategy. After removing the literature irrelevant to this topic (n = 439), 767 studies were identified as eligible, including articles (n = 534) and reviews (n = 233). We discovered a notable increasing trend in global publications in the overlapping fields of circRNAs and CVDs, from 1 publication in 2010 and 1 in 2015 to 190 publications in 2022. By the time we completed our research study, the number of studies published in 2023 was 86. The specific search formula is reported in Fig. 1.

**Fig. 2:**
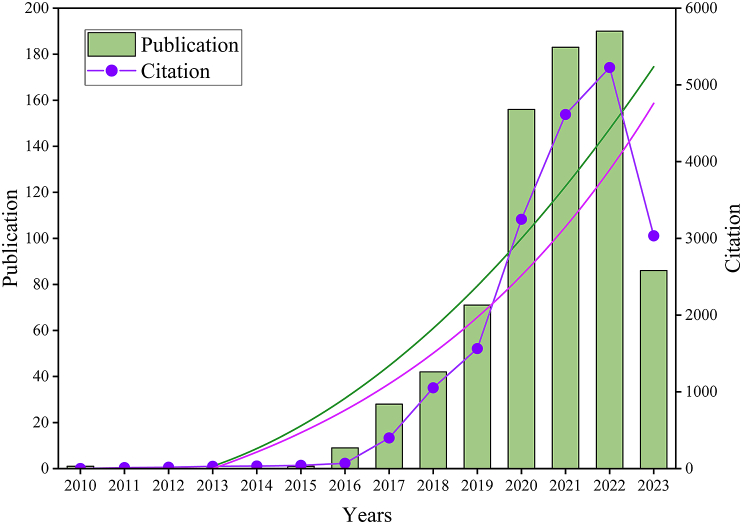
Trends in the growth of publications worldwide from 2010 to 2023.

Fig. 2 indicates that after the article published in 2010, there were no relevant publications between 2011 and 2014 (Fig. 2). The count of publications showed a remarkable yearly increase between 2015 and 2022, with a rapid burst from 2015 to 2019, while there was a slight decrease in the growth rate from 2020 to 2022. The most noticeable increase was found to have occurred from 2019 to 2020. The articles we obtained in this study were published before July 31, 2023. We speculate that the number of publications in 2023 is equal or exceeds that in 2022. An upward trend was observed in the yearly increase in citation number, which corresponded to that of the number of publications. This indicates that the overlap between these two fields has gradually attracted the attention of researchers.

### 3.2 Co-authorship: countries/regions and institutions

**Fig. 3:**
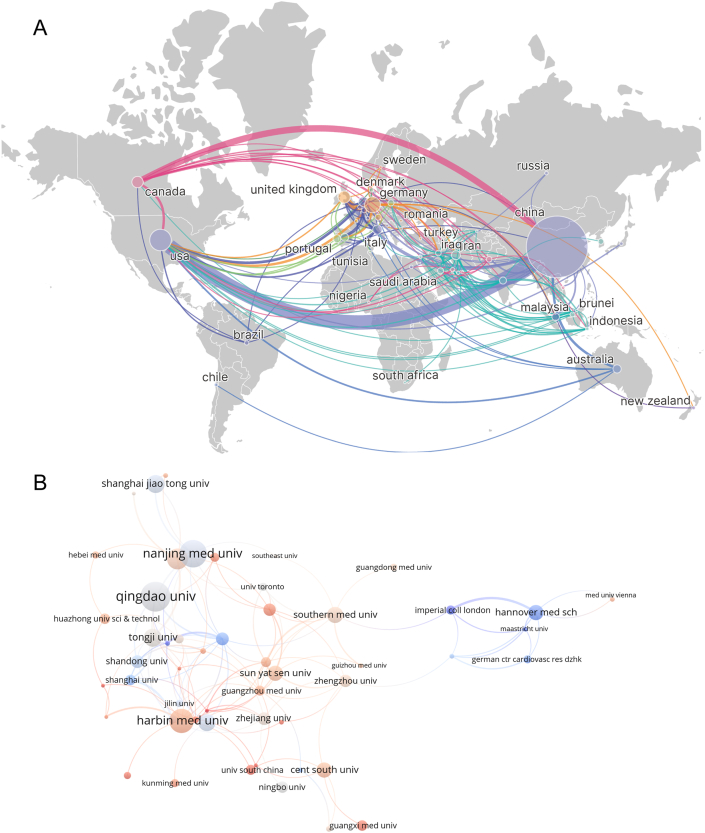
The collaboration of countries/regions and institutions in the field of circRNA in CVDs. (A) Collaboration map of the publications and co-occurrence network of countries/regions. (B) Institutional cooperation/contributions to publications.

Various countries and institutions worldwide contributed to the publications related to the role of circular RNA in cardiovascular diseases from 2010 to 2023. Table 1 and Fig. 3A showcase the number of publications and cooperation instances relative to the 46 countries that contributed to the field. China produced the greatest number of publications (621 documents), corresponding to 80.96 % of the total, a marked difference compared with other countries. Following China, most studies were published in the United States (76, 9.91 %), Germany (41, 5.35 %), England (24, 3.13 %), and Canada (21, 2.74 %). Correspondingly, Chinese studies also presented the highest number of citations, i.e., 12,928, which is more than four times that of the above countries (United States: 3045 citations; Germany: 2918 citations; England: 1399 citations; Canada: 1646 citations). The number of citations of the remaining countries aggregated in Table 1 was no more than 1000 and was thus not further investigated. These results indicate that China (620 articles, 12,928 citations, and 3700 total link strength) has played a predominant role in the contribution to publications on the application of circRNA in CVDs (Fig. 3).

**Table 1:** Top 10 countries and institutions that contributed to publications of CircRNA in CVD.

Top 10 countries that contributed to publications of CircRNA in CVD						
Rank	Country	Document	Percentage (%)	Citation	Citation/document	Total link strength
1	China	621	80.96	12,928	20.82	96
2	United States	76	9.91	3045	40.07	87
3	Germany	41	5.35	2918	71.17	50
4	England	24	3.13	1399	58.29	39
5	Canada	21	2.74	1646	78.38	36
6	Netherlands	15	1.96	877	58.47	34
7	Italy	14	1.83	541	38.64	15
8	Iran	13	1.69	87	6.69	31
9	Australia	11	1.43	255	23.18	12
10	Portugal	10	1.30	186	18.60	11
10	Spain	10	1.30	173	17.30	14
Top 10 institutions that contributed to publications of CircRNA in CVD						
Rank	Institution (Country)	Document	Percentage (%)	Citation	Citation/document	Total link strength
1	Qingdao University (China)	37	4.82	1892	51.14	10
2	Nanjing Medical University (China)	34	4.43	755	22.21	17
3	Harbin Medical University (China)	30	3.91	668	22.27	16
4	Fudan University (China)	26	3.39	625	24.04	20
5	Shanghai Jiao Tong University (China)	22	2.87	498	22.64	7
6	Tongji University (China)	22	2.87	583	26.50	25
7	Capital Medical University (China)	21	2.74	486	23.14	7
8	Southern Medical University (China)	20	2.61	770	38.50	17
9	Central South University (China)	19	2.48	398	20.95	5
10	Hannover Medical School (Germany)	19	2.48	1272	66.95	16
10	Sun Yat-sen University (China)	19	2.48	662	34.84	18

The top ten most productive institutions are displayed in Table 1. VOSviewer was applied to cluster the 53 collaborating institutions selected from a total of 58 institutions with no less than five publications, and a cluster diagram was thus created, as shown in Fig. 3B. The leading institution in number of publications was found to be Qingdao University (37 documents), followed by Nanjing Medical University (34 articles), Harbin Medical University (30 articles), and Fudan University (26 articles). Tongji University was found to have worked the most closely with other institutions, with a total link strength of 25, followed by Fudan University (20). Except for one German institution, the top 10 institutions by publication number were all from China. As can be seen from the table, there were no obvious numerical gaps between the top 10 institutions. Given that most of the cooperating institutions belonged to the same country, cross-national institutional cooperation should be increased.

### 3.3 Analysis of journals and co-cited journals

The 10 most productive journals and co-cited journals on the role of circRNAs in CVDs are ranked in Table 2. The top three journals in terms of number of publications were *Frontiers in Genetics* (24 articles), *Frontiers in Cardiovascular Medicine* (23 articles), and *Molecular Therapy—Nucleic Acids* (22 articles), each of which had more than 20 studies. According to the H-index of the top 10 most productive journals, *Molecular Therapy—Nucleic Acids* ranked first, which means that it published the most studies with high impact (H-index of 14). The *Journal of Cellular and Molecular Medicine* (H index of 10), the only other journal with an H-index of no less than 10, ranked second. With regard to total link strength, *Frontiers in Genetics* (1755 citations and 166,467 total link strength), *Frontiers in Cardiovascular Medicine* (1426 citations and 130,333 total link strength), *Molecular Therapy—Nucleic Acids* (1260 citations and 118,516 total link strength), *International Journal of Molecular Sciences* (1089 citations and 108,851 total link strength), and *Journal of Cardiovascular Pharmacology* (1046 citations and 103,478 total link strength) featured the most co-citations, along with others with a total link strength greater than 100,000 (Table 2).

**Table 2:** Top 10 journals and co-cited journals by papers of CircRNA in CVD.

Rank	Journal	Document	Total citation	Citation/Document	Total link strength	IF2022	H-index	Rank	Co-cited journal	Citations	Total link strength	IF2022
1	frontiers in genetics	24	270	11.25	8057	3.7	9	1	Circulation Research	1755	166,467	20.1
2	frontiers in cardiovascular medicine	23	142	6.17	15,263	3.6	6	2	nature	1426	130,333	64.8
3	molecular therapy-nucleic acids	22	556	25.27	18,271	8.8	14	3	circulation	1260	118,516	37.8
4	international journal of molecular sciences	17	189	11.12	19,886	5.6	8	4	cell	1089	108,851	64.5
5	journal of cardiovascular pharmacology	15	105	7.00	7455	3	7	5	plos one	1046	103,478	3.7
6	journal of cellular and molecular medicine	14	304	21.71	10,723	5.3	10	6	nucleic acids research	961	80,761	14.9
7	Bioengineered	12	97	8.08	3134	4.9	7	7	molecular cell	941	91,000	16
8	journal of clinical laboratory analysis	11	92	8.36	4558	2.7	6	8	Scientific Reports	896	88,438	4.6
9	non-coding rnas in cardiovascular diseases	11	83	7.55	9500	3.65	6	9	nature communications	893	88,753	16.6
10	oxidative medicine and cellular longevity	11	161	14.64	5774	7.31	6	10	cardiovascular research	791	75,401	10.8

**Fig. 4:**
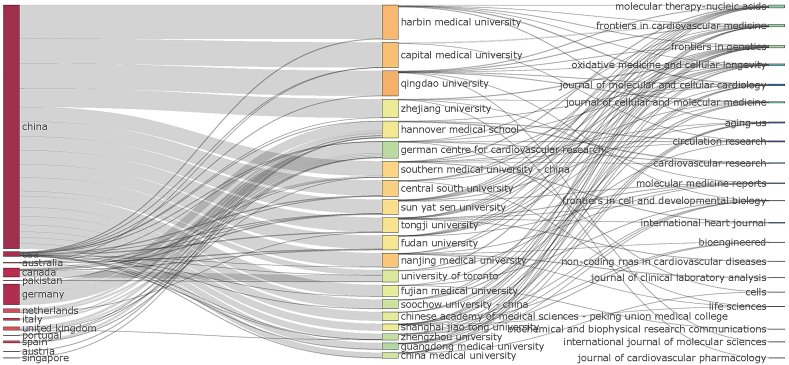
The relationship of the countries, institutions, and journals that produced articles in an alluvial flow map that was based on R for circRNA in CVDs.

The bonds among countries, regions, institutions, journals, and co-cited journals based on a three-field plot for the studied research field is shown in Fig. 4. China was associated with a maximum of 18 institutions and was closely followed by the United States. Notably, as can be seen in the first two columns of the chart, among the top six dominant institutions, Harbin Medical University, Capital Medical University, Qingdao University, and Zhejiang University mainly concentrated on domestic research, while Hannover Medical School and the German Centre for Cardiovascular Research participated more in international cooperation. Regarding the collaborations between institutions and journals, as we can see from the last two columns of the chart, the six journals with the most connections were found to be *Molecular Therapy—Nucleic Acids*, *Frontiers in Genetics*, *Frontiers in Cardiovascular Medicine*, *Oxidative Medicine and Cellular Longevity*, *Journal of Molecular and Cellular Cardiology*, and *Journal of Cellular and Molecular Medicine* (Fig. 4).

### 3.4 Analysis of authors and co-authorship

The top ten authors in terms of contribution to research on the role and mechanisms of circRNAs in CVDs are listed in Table 3. Thomas (19 documents, 1271 citations, and H-index of 16), Zhang and Yuyang (14 documents, 499 citations, and H-index of 6), and Wang and Jianxun (12 documents, 978 citations, and H-index of 6) were the most prolific authors among all the authors studied. Wang and Kun were the authors with the most citations per article among the top ten authors in this field, with an average citation number of 124.38, thus indicating that the articles they published hold high scientific value (Table 3).

**Table 3:** Top 10 authors and co-cited authors in the field of CircRNA in CVD.

Rank	Author	Document	Citation	Citation/Document	H-index
1	Thum, Thomas	19	1271	66.89	16
2	Zhang, Yuyang	14	499	39.21	6
3	Wang, Jianxun	12	978	81.50	6
4	Li, Peifeng	11	1183	107.55	10
5	Jia, Enzhi	10	124	12.40	5
6	Yang, Burton B.	9	1046	116.22	8
7	Wang,Kun	8	995	124.38	6
7	Zhang, Lina	8	116	14.50	6
9	Bar, Christian	7	419	59.86	6
9	Ge, Junbo	7	160	22.86	4
9	Ding, Wei	7	287	41.00	4
9	Du, william W.	7	862	123.14	6
9	Zheng, shuying	7	116	16.57	6
9	He, Shu	7	23	3.29	3

In contrast, Wang and Kun (460 citations and 15,868 total link strength) were the most high-yielding co-cited authors and were also among the top 10 most productive authors (Table 4). Wang and Kun's first study in this field was published under the title “A circular RNA protects the heart from pathological hypertrophy and heart failure by targeting miR-223” [41] in the *European Heart Journal* in 2016. Their research has since focused on circRNAs in terms of attenuating myocardial ischemia/reperfusion injury by inhibiting autophagy [42], apoptosis [43], and ferroptosis [44].

**Table 4:** Top 10 co-cited authors in the field of CircRNA in CVD.

Rank	Author	Citations	Total link strength
1	wang, k	460	15,868
2	hansen, tb	366	12,754
3	zhang, y	354	14,378
4	memczak, s	352	10,762
5	du, ww	318	13,723
6	jeck, wr	313	11,384
7	salzman, j	224	8983
8	holdt, lm	215	8492
9	wang, y	194	6949
10	chen, ll	178	5889

### 3.5 Analysis of references and co-cited references

The top 10 references by citation number are listed in Table 5. There were six articles and four reviews, a roughly balanced assortment. Holdt et al. (2016) (747 citations), Burd et al. (2010) (659 citations), and Wang et al. (2016) (641 citations) were the most prolific references among all, with the highest numbers of citations listed in our result table. The targeted signaling pathways focused upon in these three studies differed, which implies that there are great research prospects in this field from a molecular biology perspective.

**Table 5:** The top 10 references and co-cited references based on the number of citations.

Top 10 references based on the number of citations							
Rank	Citations	Title	First Author	Document type	Year	Journal	IF (2022)
1	747	Circular non-coding RNA ANRIL modulates ribosomal RNA maturation and atherosclerosis in humans	Holdt, Lesca M.	Article	2016	Nature Communications	16.6
2	695	Expression of Linear and Novel Circular Forms of an INK4/ARF-Associated Non-Coding RNA Correlates with Atherosclerosis Risk	Burd, Christin E.	Article	2010	Plos Genetics	–
3	641	A circular RNA protects the heart from pathological hypertrophy and heart failure by targeting miR-223	Wang, Kun	Article	2016	European Heart Journal	39.3
4	496	Foxo3 circular RNA promotes cardiac senescence by modulating multiple factors associated with stress and senescence responses	Du, William W.	Article	2017	European Heart Journal	39.3
5	325	Circular RNA in cardiovascular disease	Altesha, M-Ashraf	Review	2019	Journal Of Cellular Physiology	5.6
6	310	The emerging landscape of circular RNA in life processes	Qu, Shibin	Review	2017	Rna Biology	4.1
7	310	The Circular RNA Cdr1as Promotes Myocardial Infarction by Mediating the Regulation of miR-7a on Its Target Genes Expression	Geng, Hai-Hua	Article	2016	Plos One	3.7
8	270	A novel identified circular RNA, circRNA_010,567, promotes myocardial fibrosis via suppressing miR-141 by targeting TGF-beta 1	Zhou, Bing	Article	2017	Biochemical And Biophysical Research Communications	3.1
9	261	Circulating Noncoding RNAs as Biomarkers of Cardiovascular Disease and Injury	Viereck, Janika	Review	2017	Circulation Research	20.1
10	259	A Circular RNA Binds To and Activates AKT Phosphorylation and Nuclear Localization Reducing Apoptosis and Enhancing Cardiac Repair	Zeng, Yan	Review	2017	Theranostics	12.4
Top 10 co-cited references based on the number of citations							
**Rank**	**Citations**	**Title**	**First Author**	**Document type**	**Year**	**Journal**	**IF(2022)**
1	306	Circular RNAs are a large class of animal RNAs with regulatory potency	Sebastian Memczak	Article	2013	Nature	64.8
2	265	Natural RNA circles function as efficient microRNA sponges	Hansen, Thomas B.	Article	2013	Nature	64.8
3	222	A circular RNA protects the heart from pathological hypertrophy and heart failure by targeting miR-223	Wang, Kun	Article	2016	European Heart Journal	39.3
4	200	Circular RNAs are abundant, conserved, and associated with ALU repeats	Jeck, William R.	Article	2013	RNA	4.5
5	153	The Circular RNA Cdr1as Promotes Myocardial Infarction by Mediating the Regulation of miR-7a on Its Target Genes Expression	Geng, Hai-Hua	Article	2016	PLOS ONE	3.7
6	147	Circular non-coding RNA ANRIL modulates ribosomal RNA maturation and atherosclerosis in humans	Holdt, Lesca M.	Article	2016	Nature Communications	16.6
7	147	Exon-intron circular RNAs regulate transcription in the nucleus	Li, Zhaoyong	Article	2015	Nature Structural & Molecular Biology	16.8
8	140	circRNA Biogenesis Competes with Pre-mRNA Splicing	Ashwal-Fluss, Reut	Article	2014	Molecular Cell	16
9	136	Foxo3 circular RNA promotes cardiac senescence by modulating multiple factors associated with stress and senescence responses	Du, William W.	Article	2017	European Heart Journal	39.3
10	128	Circular Intronic Long Noncoding RNAs	Zhang, Yang	Article	2013	Molecular Cell	16

The top 10 co-cited references on the topic of the role of circRNAs in CVDs are also displayed in Table 5. Similarly, as we can see from the table, Sebastian et al. (2013), Hansen et al. (2013), and Wang et al. (2016) ranked at the top. Interestingly, the top two articles were published in the world's earliest-established international scientific and technological journals, as well as in one of the most authoritative academic journals in the world, *Nature*; in these studies, the authors mainly investigated the properties of circRNAs and provided a solid research foundation for the field of circRNA in CVDs. The third-ranked article, on the other hand, was identified as the first to explore the field, representing the prelude to in-depth discussions and investigations. Lastly, there were no reviews among the top 10 co-cited references.

**Fig. 5:**
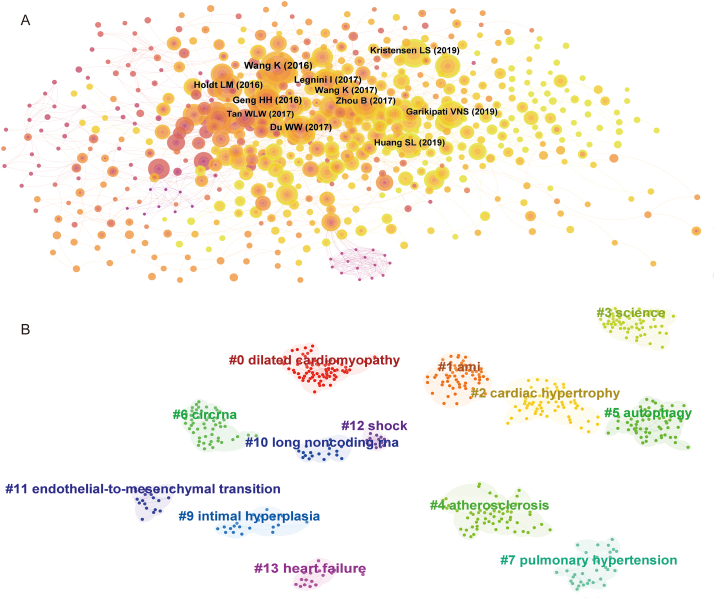
The collaboration of authors and co-cited references in the field of circRNA in CVDs. (A) Cooperation network among the co-cited references in the studies of circRNA in CVDs. (B) A cluster of the co-cited references in the studies of circRNA in CVDs.

**Fig. 6:**
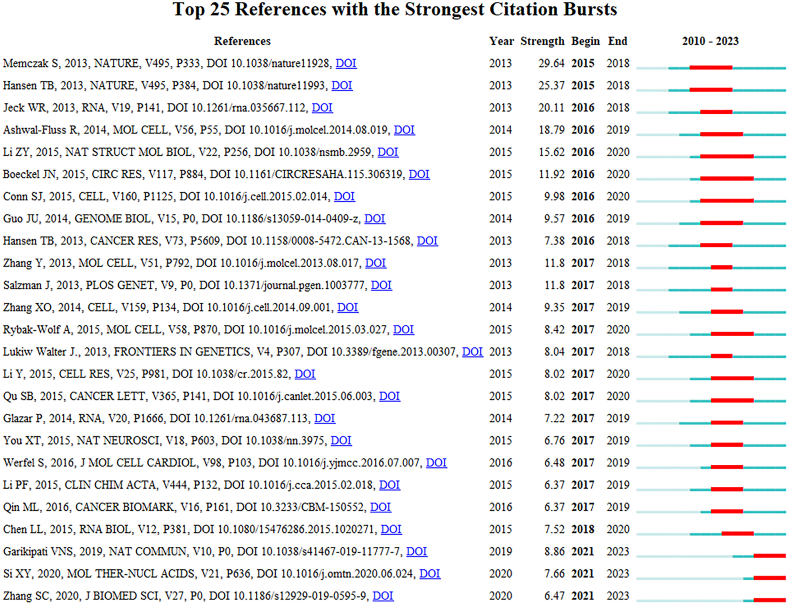
The top 25 references with the strongest citation bursts, based on CiteSpace, that were involved in circRNA in CVDs between 2010 and 2022. The blue line represents the time from its first appearance to 2022, and the red line represents the burst time.

We used CiteSpace to extract the co-cited references, as shown in Fig. 5A, and then cluster-analyzed them, as shown in Fig. 5B, obtaining 13 clusters: 0) dilated cardiomyopathy; 1) AMI; 2) cardiac hypertrophy; 3) science; 4) atherosclerosis; 5) autophagy; 6) circRNA; 7) pulmonary hypertension; 9) intimal hyperplasia; 10) long noncoding RNA; 11) endothelial-to-mesenchymal transition; 12) shock; and 13) heart failure (Fig. 5). The clusters with the highest burst strength were found to be dilated cardiomyopathy, AMI, and cardiac hypertrophy. These areas, to some extent, indicate the main research focus in the overlapping fields of circRNAs and CVDs. The top 25 co-cited references with the strongest citation bursts are depicted in Fig. 6. These references were spread over a specific period and accordingly reflected the hotspots which characterized that time. The first citation burst occurred between 2013 and 2018, as shown in Fig. 6, and the latest one was recorded from 2020 to the present. The greatest burst strength was that of the studies by Memczak et al. (2013) (29.64 strength), Hansen et al. (2013) (25.37 strength), and Jeck et al. (2013) (20.11 strength). Furthermore, Garikipati et al. (2019), Si et al. (2020), and Zhang et al. (2020) have received increasing attention in recent years (Fig. 6).

### 3.6 Keyword analysis

The top 20 most frequent keywords related to circRNAs in CVDs are listed in Table 6 (Table 6). CircRNA (508 occurrences and 3293 total link strength) was the keyword with the greatest number of occurrences, followed by miRNA (213 occurrences and 1614 total link strength), expression (168 occurrences and 1311 total link strength), and ncRNA (137 occurrences and 1107 total link strength). Among the top twenty keywords, myocardial infraction (114 occurrences and 840 total link strength), cardiovascular disease (92 occurrences and 790 total link strength), atherosclerosis (90 occurrences and 569 total link strength), heart (87 occurrences and 602 total link strength), and heart failure (68 occurrences and 530 total link strength) were the keywords that are most closely related to CVDs (Table 6).

**Table 6:** Top 20 frequency keywords related to CircRNA in CVD.

Ranking	keyword	occurrences	total link strength	Ranking	keyword	occurrences	total link strength
1	circRNA	508	3293	11	atherosclerosis	90	569
2	miRNA	213	1614	12	heart	87	602
3	expression	168	1311	13	cancer	72	506
4	ncRNA	137	1107	14	mechanism	72	519
5	apoptosis	131	905	15	heart-failure	68	530
6	biomarker	126	992	16	identification	68	566
7	myocardial infarction	114	840	17	biogenesis	64	505
8	lncRNA	112	926	18	cell	63	413
9	proliferation	98	712	19	disease	50	334
10	cardiovascular disease	92	790	20	ceRNA	46	328

**Fig. 7:**
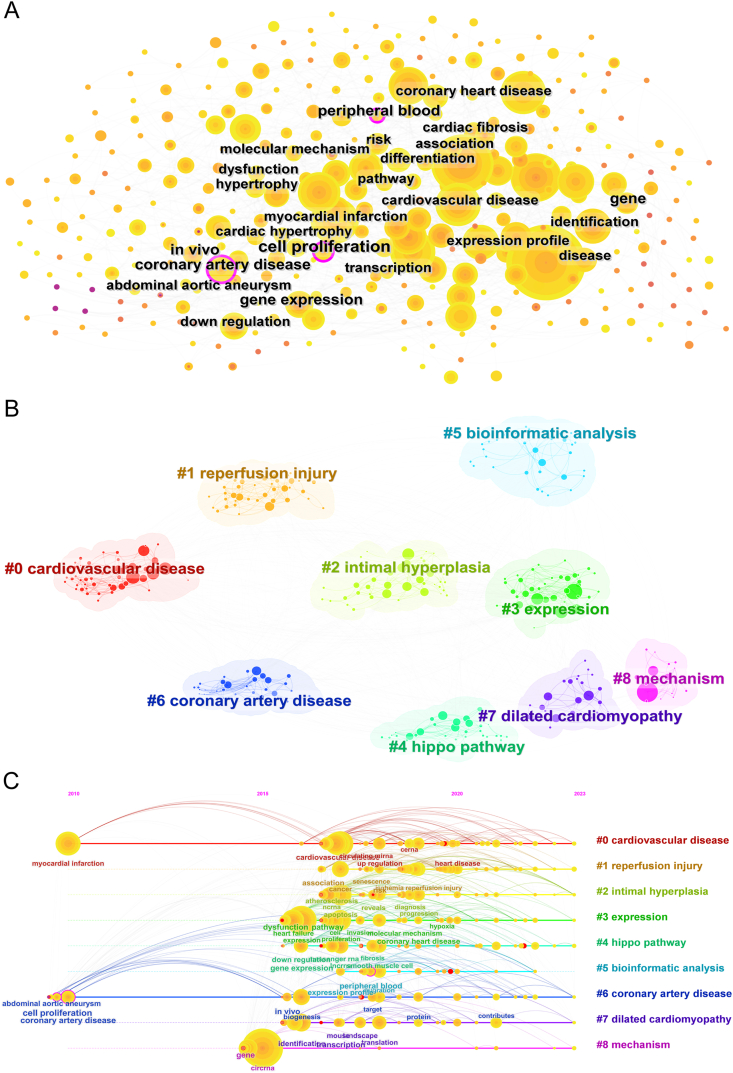
Keyword-related mapping in studies in the field of circRNA in CVDs. (A) Map of the keywords in the studies of circRNA in CVDs. (B) Subcategories of the main keywords that were utilized in the studies related to circRNA in CVDs. (C) A timeline view of keywords, based on CiteSpace, that are related to circRNA in CVDs.

CiteSpace software was employed to group the keywords and references to obtain a timeline for the keywords after clustering (Fig. 7). The associations among the keywords are demonstrated in a map (Fig. 7A). As shown in Fig. 7B, there were nine clusters: 0) cardiovascular disease; 1) reperfusion injury; 2) intimal hyperplasia; 3) expression; 4) hippo pathway; 5) bioinformatic analysis; 6) coronary artery disease; 7) dilated cardiomyopathy; and 8) mechanism. According to the timeline (Fig. 7C), though the arrangement of the keywords seems to be dispersive, we found that myocardial infraction, abdominal aortic aneurysm, cell proliferation, and coronary artery disease indicated the initial areas of interest; afterwards, the focus shifted towards the keywords grouped in the mechanism cluster. Finally, the keywords relevant to expression indicated that this topic held the attention of researchers for the longest period of time.

**Fig. 8:**
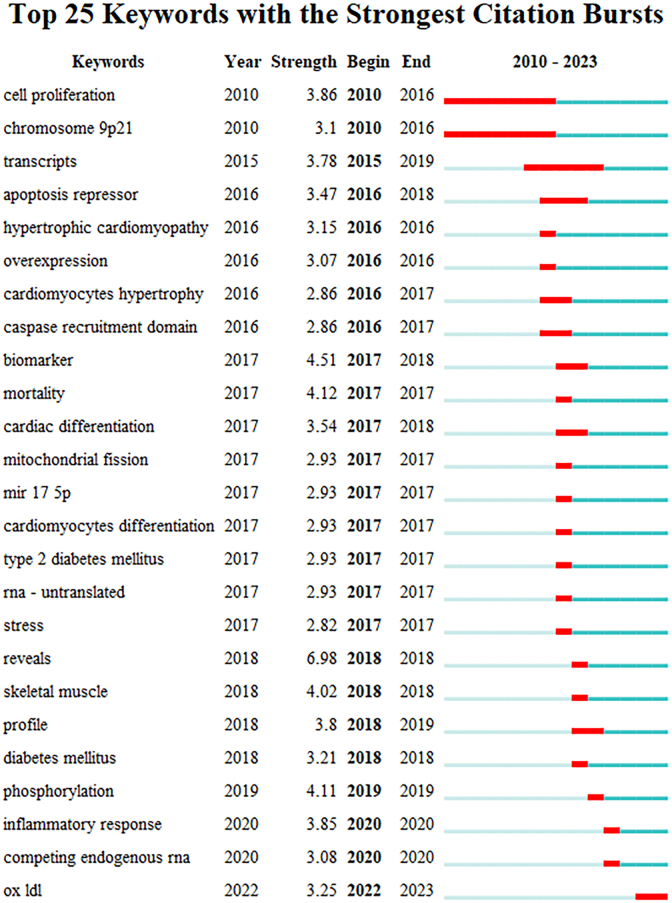
The top 25 keywords related to circRNA in CVDs with the strongest bursts, as determined via CiteSpace.

The top 25 keywords with the strongest citation bursts, based on CiteSpace, are listed in Fig. 8. The longest burst periods were relative to cell proliferation and chromosome 9p21, with both having lasted from 2010 to 2016. In contrast, the highest burst strength related to circRNAs in CVDs was from reveals (strength = 6.98), biomarkers (strength = 4.51), mortality (strength = 4.12), and phosphorylation (strength = 4.11). In addition, inflammatory response (2020), competing endogenous RNA (2020), and ox-LDL (2020) were found to be currently receiving the most attention (Fig. 8).

**Fig. 9:**
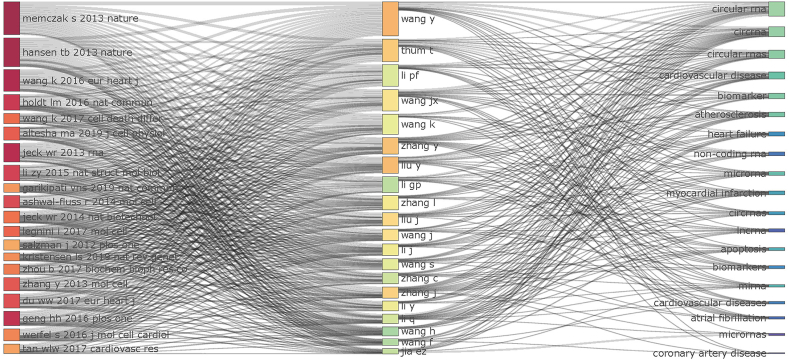
The relationships of the top 20 co-cited reference, author, and keyword evolutions based on an alluvial flow map by R.

The associations and connections among the top 20 co-cited references, authors, and keywords in the field of circRNAs in CVDs are reported in Fig. 9 as an alluvial flow map. It is evident that the 20 most co-cited references were more closely related to research on the role of circRNAs in various types of CVDs (Fig. 9).

## 4 Discussion

Our study was a bibliometric analysis conducted by using R-language based Bibliometrix Package with the aims to summarize the latest progress, identify research hotspots, and—most importantly—discuss future development trends in the field of circRNAs in CVDs. It is meaningful to provide valuable insights into research hotspots and trends with tables; graphically represented results; and statistics on countries and regions, institutions, authors, references, and keywords in this area.

### 4.1 Bibliometric information

As Fig. 2 shows, there was a yearly increasing trend in the number of publications. Articles for the second half of 2023 were not included due to the criterion for publication collection; as such, the curve with dots peaked in the year 2022. Furthermore, the figure also shows that the growth rate of citations was greater than that of publications.

CircRNAs were discovered in the 1970s. In recent years, with the evolution and popularization of technologies such as second-generation sequencing, various properties of cyclic RNAs have been revealed [[45], [46], [47]]. This brand-new field of research has also grown rapidly in a short period of time. Between 2010 and 2015, the research on circRNAs in CVDs did not develop significantly. Since 2016, this field has received significant attention and has witnessed a quick increase in the number of related research reports. The study of circRNAs is now an established research direction, and it is believed that it will provide enlightening insights into the field of CVDs in the future [48,49].

Thomas Thum, who was awarded the Paul Martini Prize in 2021, was found to be the most prolific author (19 publications and H-index of 16). He and his team have been mainly dedicated to the research of miRNA as a potential treatment for heart failure. Chinese scholars published the most studies, to a degree of nine times more than the second-ranked country, the US, and nine-tenths of the most productive institutions were found to be Chinese. This may be due to the large Chinese population. In addition, China has the highest CVD morbidity and mortality rates in the world, which may also account for this result. Therefore, it is not that surprising that Chinese scholars are the most present and active in the field of circRNA in CVDs. Furthermore, among the top ten most cited articles, the sum of citations of research studies by Chinese scholars accounted for 35 per cent of the aggregate of the top ten citations (1531/4314) (Table 5), with their citations/studies ranking eighth (Table 1). To some extent, this indicates that the influence of Chinese scholars is low and that more effort is required on their part for achieving greater representation among the most cited studies in the field of circRNA in CVDs.

In addition, as shown in Table 3, Table 4, Table 5, while the journal institutions publishing circRNA-related studies were mostly based in China, different findings were obtained by investigating the citation number. The articles with the largest citation number were mainly from cutting-edge journals such as *Nature* and *Cell*. The authors of these journals mainly consist of non-Chinese scholars, with a great share being from the United States, Germany, Australia, Italy, and Canada. This suggests that although most circRNA-related articles are from China, to some extent, there is a lack of high-quality Chinese studies, a fault which can be seen as the major reason for their low representativeness. One possible reason for this phenomenon is that rather than concentrating on the specific mechanisms of circRNAs, Chinese authors have focused more on the biological role of circRNAs in diseases.

Overall, as demonstrated by the bibliometric results, research on circRNA in CVDs is still booming, with researchers from various countries advancing the field in various aspects and making joint contributions to the rapid research development that will likely continue in the future.

### 4.2 Research hotspots and future directions

#### 4.2.1 The molecular mechanisms and role of circRNAs

The top two articles with the most citations both discussed the relationship between circANRIL, which is derived from long non-coding RNAs, and atherosclerosis [50,51]. The interaction between circRNAs and proteins is an important molecular mechanism in terms of the role of these molecules. On one hand, the direct binding of circRNAs to proteins can support protein function [52]. On the other hand, the combination of circRNAs and proteins can disrupt the subcellular localization or functioning of proteins, which in turn results in differences in cellular functioning [53]. The most cited article revealed that circANRIL binds to the pescadillo homologue 1 (PES1) protein in order to regulate precursor ribosomal RNA (pre-rRNA) maturation, which, in turn, can regulate atherosclerosis [50].

Signaling pathways play an indispensable role in cellular physiological activities, as specific cellular functions can be adjusted through the regulation of key proteins. For instance, it has been proven that the Hippo signaling pathway can regulate cell proliferation and apoptosis; specifically, studies have shown that the Hippo signaling pathway is involved in the regulation of several cardiovascular physio-pathological processes, including cardiovascular development, hypertrophy, apoptosis, autophagy, angiogenesis, and regeneration [54,55]. The regulation of the Hippo signaling pathway is also thought to reduce CVD risk [56]. Shohei et al. reviewed the role of this pathway in cardiomyocyte growth and found that Yap plays an important role in heart regeneration after myocardial infarction [57]. Furthermore, Shao et al. also discovered that circ-CDR1 could activate the Hippo pathway and that CDR1as knockdown could ameliorate apoptosis induced by diabetic cardiomyopathy [58].

In recent years, the role of circRNAs in crucial cellular signaling pathways has also been greatly investigated (Fig. 7) [59,60]. For instance, the most cited paper reported that circ-Amotl1, a circular RNA, can physically bind with PDK1 and AKT1, thus promoting the protective nuclear translocation of pAKT, which provides protection against Adriamycin-induced cardiomyopathy [61]. In addition, Wang et al. found that circ_0002984 downregulation inhibits cell proliferation and migration through the regulation of the miR-181b-5p/VEGFA axis and the phosphatidylinositol 3-kinase-AKT pathway in oxygenated low-density-lipoprotein-stimulated VEGFA, which provides a new route for the exploration of the pathogenesis of AS [62]. Furthermore, the research study by Li et al. found that circARAP1 promotes MI/RI- and H/R-induced cardiomyocyte injury in mice by activating Wnt/β-linker signaling through the regulation of the miR-379-5p/KLF9 axis [63]. Additionally, Chen et al. reviewed the critical role of circRNAs in cancer, mainly by focusing on the relationship between the expression of PI3K-AKT-related circRNAs and clinicopathological features, as well as discussing the important role of circRNAs in cancer diagnosis, prognosis, and treatment [64]. Moreover, Huang et al. investigated the role of circ_SMG6 in myocardial ischemia/reperfusion and found that it can worsen myocardial I/R injury; in addition, they found that neutrophil recruitment is promoted through the miR-138-5p/EGR1/TLR4/TRIF signaling pathway, which provides a new direction for research on the treatment of myocardial I/R injury [65]. Furthermore, Jin et al. investigated the role of circRbms1 in AMI and found that circRbms1 is involved in myocardial I/R injury through the regulation of the miR-92a/BCL2L11 signaling pathway [66].

The regulation of gene expression is also an important mechanism in circRNAs, which can modulate downstream target gene expression by attaching themselves to miRNAs. The adsorption of endogenous miRNAs by cyclic RNA sponges was first reported by Memczak et al., in 2013 [67,68], with the regulation of circRNAs through miRNAs attracting great interest ever since. This has also been reported in studies in CVDs (Fig. 8). In the third most cited article, the authors revealed that circ-heart-related circRNAs (HRCRs) can act as endogenous miR-223 sponges that inhibit cardiac hypertrophy and heart failure [41]. In another one of the most cited articles, the authors revealed that Cdr1as overexpression promoted cardiomyocyte apoptosis and increased infarct size, which could be reversed with miR-7a overexpression, thus broadening the current treatment for myocardial infarction [69]. In the seventh most cited article, i.e., the study by Zhou et al., it was indicated that circRNA_010,567 silences upregulates miR-141, downregulates TGF-β1 expression, and inhibits fibrosis-associated proteolytic excision in CFs (including Col I, Col III, and α-SMA), which represents a novel role of circRNAs in the pathogenesis of central myofibrillar fibrosis [70]. So far, the regulation of gene expression by circRNA sponges through their attachment to endogenous miRNAs remains the most widely reported mechanism, and this trend may be sustainable. However, it is worth noting that since the expression level of circRNAs is generally low in mammals and the number of binding sites in miRNAs is relatively low, the idea of using circRNAs to control the stability and quantity of miRNAs so as to achieve measurable effects should be considered carefully. In addition, circRNAs can also regulate gene expression by competing with homologous mRNAs to bind to endogenous pre-mRNAs [71]. However, this mechanism has not been reported in CVDs.

Since they were discovered, circRNAs were considered not to possess a coding function for a long time. Their ability to code proteins was first revealed in 2017 [72], and research on polypeptide translation by circRNAs has been extensive ever since. Currently, it has become popular to study the association between these molecules and cancer [16]. Though studies on the polypeptide translational function of circRNAs in CVDs are still lacking (Fig. 7C) [29], given increasing research on the peptide-coding function, the maturation of synthesis and translation techniques in vitro, and the rapid development of circRNA vaccines, the study of circRNA in CVDs is also promising and large in scope.

Furthermore, the discovery of the interaction between circRNA and genomic DNA has also overcome the limitations of past research. For example, Mo Chen reported in detail the causes of leukemia gene translocations, in which the role of circRNAs was highlighted [73]. In addition, Chen et al. found that low m2A methylation levels of circGPATCH2L promote the accumulation of DNA damage and apoptosis and aggravate intervertebral disc degeneration (IVDD), whereas m6A-methylated circGPATCH2L is readily degraded and could be a therapeutic target for IVDD [74]. Furthermore, in their study, Yao et al. reported that B [a]P can upregulate circ_00035,526 with a modification through m6A, thereby promoting DNA damage [75]. Moreover, Ajit et al. analyzed and commented on the article by Conn et al. in *Cancer Cell*, concluding that Conn et al. showed a novel functional role for circRNAs as drivers of genomic instability and asserting that the measurement of cancer circRNAs could help to better track disease progression [76]. Hopefully, this will be also applied to studies on other diseases in the future.

In summary, the molecular mechanism that underpins the role of circRNAs in CVDs is a current research hotspot.

#### 4.2.2 Applications of circRNAs in CVDs

The abundant expression and high stability of circRNAs, as well as their specific expression in tissue and disease, make them ideal and promising biomarkers [77], as already extensively studied in terms of diagnosing diseases or indicating disease progression or prognosis, especially in cancer [78]. In particular, circRNAs have been regarded as biomarkers in CVDs (Fig. 8). For example, Yang et al. summarized the applications of circRNAs in the non-cancer field, finding evidence to suggest that the detection of various circRNAs in peripheral blood could be predictive of CVDs such as atherosclerosis, myocardial infarction, and atrial fibrillation [79]. Zhang et al. also discussed circRNAs’ potential role in clinical applications as biomarkers and therapeutic targets, as well as in angiogenesis [80]. Priscilla et al. reported the promising role of circRNAs as biomarkers in hypertension [81]. Furthermore, Wang et al. summarized the development and function of circRNAs in CVDs, and noted the possibility for circRNAs to be used as biomarkers in non-toxicity heart diseases [82]. Chen et al. proposed an innovative perspective on the circRNA–miRNA–mRNA interaction network and on circRNAs acting as novel markers in CVDs in diagnosis and treatment [83]. Ahmed et al. also reviewed the latest research results and emphasized the great potential of circRNAs as biomarkers for the early detection of CVDs [84]. David et al. found evidence of the downregulation of hsa_circ_0001445 in extracellular vesicles secreted by human coronary smooth muscle cells in atherosclerosis, which suggests that plasma hsa_circ_0001445 could be a biomarker of coronary atherosclerosis [85]. Bao et al. proved that the expression level of hsa_circ_0037,911 in patients with essential hypertension was significantly higher than that in healthy subjects and that of has-miR-637 significantly lower, indicating that the joint action of hsa_circ_0037,911 and has-miR-637 may be a promising biomarker for the early diagnosis of essential hypertension [86].

In-depth research has been conducted aiming to discover mechanisms and potential therapy for CVDs [[87], [88], [89], [90]]. In recent years, RNA therapy is a brand-new treatment that has rapidly transitioned from fundamental experiments to clinical trials [91]. Prophylactic vaccines based on circRNA therapeutic approaches have shown broad prospects for further medical research and applications [92]. On the one hand, as a part of RNA therapy, circRNA therapy performs its role in modulating gene expression [93]. On the other hand, by involving advanced technologies such as CRISPR-Cas9 or siRNA, local circRNAs can be targeted to act as biomarkers or sponging treatment in different diseases [94,95]. Some studies have suggested that circRNAs show great promise for a future role as therapeutic targets or agents in CVDs. Studies have also proven that circRNAs are of vital importance in the pathological processes of CVDs. For instance, Sheila et al. summarized the roles of ncRNAs in myocardial infarction and regeneration and reported that ircRNAs can act as microRNA sponges and subsequently regulate the expression of key signaling pathways that affect the inflammatory response or even directly regulate inflammatory markers [96]. Zhang et al. found that circRNAs could inhibit oxidative stress via the circRNA Galntl6/miR-335/Lig3 axis, suggesting that circRNA Galntl6 is a potential target for treatment aimed at preventing oxidative stress [97]. Li et al. discovered that circRNA1615 inhibits iron porphyrin deposition in cardiomyocytes by lowering the level of low-density-lipoprotein receptor-associated protein 6 via spongy miR-152-3p, thereby ameliorating the pathological process of MI [98]. Zheng et al. demonstrated that the overexpression of circRNA Samd4 reduces the size of fibrotic areas and improves cardiac function after myocardial infarction; the results of a functional analysis show that circSamd4 reduces Vdac1 expression by recruiting Vcp proteins to the mitochondria, thereby maintaining mitochondrial dynamics and reducing mitochondria-derived ROS (Fig. 8) [99]. Xu et al. showed that the downregulation of circTRRAP attenuated hypoxia-induced inflammation in human AC16 cardiomyocytes via the miR-16/MAP761K3 axis [100]. Additionally, Li et al. found that the knockdown of CircHSPG2 protects AC2 cells from hypoxia-induced injury; according to the authors, it also reduces apoptosis by modulating the miR-16/MAP1184K3 cascade response [101]. Wang et al. found that CircUSP39 promotes hypoxia/reoxygenation (H/R)-induced oxidative stress, inflammation, and miR-362-3p/TRAF3 axis-mediated apoptosis in cardiomyocytes, thereby providing a new target for the treatment of AMI [102]. In vivo experiments showed that Circ-INSR overexpression prevents and reverses Adriamycin-mediated cardiomyocyte death and improves cardiac function [103]. In summary, from the studies listed above, the same conclusion can be drawn: circRNAs could become therapeutic targets or agents in CVDs. This is expected to become a hotspot in CVD research in the future.

The role of circRNAs has been further investigated in CVDs such as dilated cardiomyopathy, acute myocardial infarction, and ventricular remodeling (Fig. 5). At present, research on cardiomyocyte regeneration in heart disease has received great attention, representing a direction in which to explore more possible treatments for CVDs [104]. For example, Huang et al. discovered that the loss of circNfix can induce cardiac regeneration after myocardial infarction, which may provide an alternative treatment for improving the prognosis after MI [105]. In recent years, the role of circRNA in cardiomyocyte regeneration has also been extensively researched (Fig. 7A).

### 4.3 Limitation

In our study, we extracted data from the WoSCC database with a validated and effective retrieval strategy, but there are limitations that should be taken into account. First of all, all of the studies included were obtained from a single database, which may have resulted in moderately biased and unbalanced initial data. Furthermore, it is inevitable that the algorithms with which we generated the results might have flaws. Moreover, due to the limitation of the time period for which we obtained the relevant articles, some contemporary articles may have been excluded from our search results. As a consequence, further analysis is still required to clarify the scientific trends and hotspots in research on circRNAs in CVDs. Nevertheless, in this bibliometric analysis, we thoroughly examined the data and offered valuable insights into the origination and development of the field of circRNA in CVDs; as such, our findings provide additional clarity on potential research hotspots and directions for future investigations.

## 5 Conclusions

**Fig. 10:**
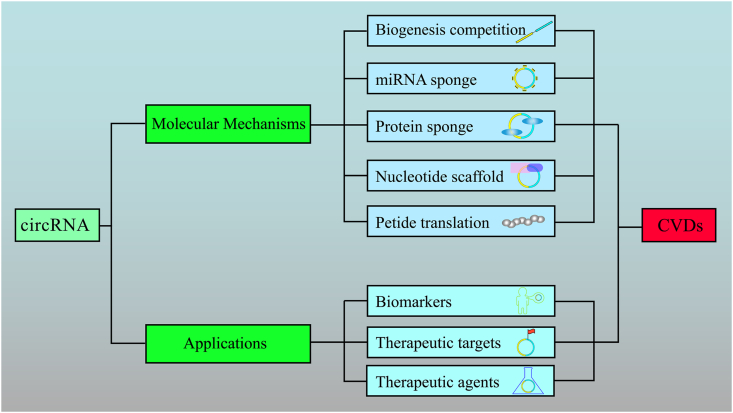
Different molecular mechanisms and potential applications that circRNAs tend to play in CVDs have been annotated in the figure. The main actional modes of circRNAs currently confirmed in cells are showed in light blue boxs, while the main applications of which are listed in purple boxes, with icons enclosed respectively.

Our study was a bibliometric analysis that assessed the results and impacts of related studies. We summarized the latest progress, identified the research hotspots, and discussed prospects in the field of circRNA in CVDs (Fig. 10). Currently, the molecular mechanisms underpinning the role of these molecules in CVDs represent a highly studied research topic. As the regulation of signaling pathways is crucial in physiological cell activities, the modulation of gene expression and the functions of coding and peptide translation are current research hotspots. Finally, promising results in CVD studies indicate that circRNAs could be used as biomarkers for diagnosing diseases or indicating the progression or prognosis of diseases.

## CRediT authorship contribution statement

**Zehui Xu:** Writing – original draft, Formal analysis, Conceptualization. **Chong Guan:** Validation, Project administration. **Ziji Cheng:** Investigation, Data curation. **Houle Zhou:** Visualization, Resources. **Wanting Qin:** Visualization. **Jiaming Feng:** Visualization. **Melisandre Wan:** Investigation. **Yihan Zhang:** Investigation. **Chengyao Jia:** Investigation. **Shuijin Shao:** Investigation. **Haidong Guo:** Writing – review & editing. **Shaoling Li:** Writing – review & editing. **Baonian Liu:** Writing – review & editing, Conceptualization.

## Declaration of competing interest

We declare that we have no financial and personal relationships with other people or organizations that can inappropriately influence our work, there is no professional or other personal interest of any nature or kind in any product, service and/or company that could be construed as influencing the position presented in, or the review of, the manuscript entitled.
